# Development and Validation of Nomograms to Predict Overall Survival and Cancer-Specific Survival in Patients With Pancreatic Adenosquamous Carcinoma

**DOI:** 10.3389/fonc.2022.831649

**Published:** 2022-03-07

**Authors:** Zhen Yang, Guangjun Shi, Ping Zhang

**Affiliations:** ^1^ Department of Hepatopancreatobiliary Surgery, Qingdao Municipal Hospital, Qingdao University, Qingdao, China; ^2^ Department of Gynecology, Qingdao Municipal Hospital, Qingdao University, Qingdao, China

**Keywords:** pancreatic adenosquamous carcinoma, nomogram, overall survival, cancer-specific survival, chemotherapy, surgery

## Abstract

**Background:**

Pancreatic adenosquamous carcinoma (PASC) is a heterogeneous group of primary pancreatic cancers characterized by the coexistence of both glandular and squamous differentiation. The aim of this study was to develop nomograms to predict survival outcomes in patients with PASC.

**Methods:**

In this retrospective study, data on PASC, including clinicopathological characteristics, treatments, and survival outcomes, were collected from the SEER database between 2000 and 2018. The primary endpoints were overall survival (OS) and cancer-specific survival (CSS). The eligible patients were randomly divided into development cohort and validation cohort in a 7:3 ratio. The nomograms for prediction of OS and CSS were constructed by the development cohort using a LASSO-Cox regression model, respectively. Besides the model performance was internally and externally validated by examining the discrimination, calibration, and clinical utility.

**Results:**

A total of 632 consecutive patients who had been diagnosed with PASC were identified and randomly divided into development (n = 444) and validation (n = 188) cohorts. In the development cohort, the estimated median OS was 7.0 months (95% CI: 6.19–7.82) and the median CSS was 7.0 months (95% CI: 6.15–7.85). In the validation cohort, the estimated median OS was 6.0 months (95% CI: 4.46–7.54) and the median CSS was 7.0 months (95% CI: 6.25–7.75). LASSO-penalized COX regression analysis identified 8 independent predictors in the OS prediction model and 9 independent risk factors in the CSS prediction model: age at diagnosis, gender, year of diagnosis, tumor location, grade, stage, size, lymph node metastasis, combined metastasis, surgery, radiation, and chemotherapy. The Harrell C index and time-dependent AUCs manifested satisfactory discriminative capabilities of the models. Calibration plots showed that both models were well calibrated. Furthermore, decision curves indicated good utility of the nomograms for decision-making.

**Conclusion:**

Nomogram-based models to evaluate personalized OS and CSS in patients with PASC were developed and well validated. These easy-to-use tools will be useful methods to calculate individualized estimate of survival, assist in risk stratification, and aid clinical decision-making.

## Introduction

Pancreatic cancer, a deadly disease with a highly metastatic potential and an unfavorable prognosis, is the fourth leading cause of cancer-related mortality in United States (1–3). Although with tremendous advances in diagnostic techniques and treatment modalities, the incidence of pancreatic cancer increased rapidly while the survival probability remains unchanged (4–6). The primary histological type of pancreatic malignancy is pancreatic ductal adenocarcinoma (PDAC) (7–9). Pancreatic adenosquamous carcinoma (PASC) is an extremely rare subtype which contains biphenotypic characteristics of both glandular and squamous differentiation as the normal pancreas is histologically devoid of squamous elements, only accounting for 0.4% to 4% of pancreatic cancer (10, 11). However, the definition of PASC remains controversial in terms of the proportion of the squamous-cell component. The exact proportion of at least 30% squamous differentiation prerequisite for diagnosis of PASC is arbitrary and subjective (12, 13). As a unique histopathological variant, PASC presents with more aggressive behaviors and more dismal prognosis compared to PDAC, which are deemed to be pathologically relevant to the squamous metaplasia (11, 14–17). Other studies demonstrate that the overall survival is similar between PASC and PDAC, even though PASCs tend to have more apparent perineural infiltration and increased lymph node involvement compared with PDAC (18). Due to the relative rarity of this malignancy, the natural history is, however, not well described. Most of the literature is mainly presented as isolated case reports or small number of case cohort studies (16, 19–23). Large population-based analyses with regard to epidemiology and clinical features of PASC are sparse. The aim of this current study was to determine epidemiological characteristics and to estimate the individualized prognosis of patients with PASC, pooling data from a population-based database and eventually developing nomograms to predict survival outcomes as well as aid clinical decision-making.

## Methods

### Study Design and Patients

In this retrospective prognostic study, clinical data and survival outcomes regarding patients initially diagnosed with PASC between 2000 and 2018 were retrieved and screened from the Surveillance, Epidemiology, and End Results (SEER) database of the National Cancer Institute (NCI). Baseline characteristics and clinicopathologic variables collected included sex, age, year of diagnosis, race, marital status, tumor characteristics, treatment details, overall survival, and cancer-specific survival. Patients whose diagnosis of PASC was confirmed by positive histology were included in the study. A total of 632 consecutive patients with complete data and follow-up information were identified. The entire cohort (n = 632) was randomly divided into development cohort (n = 444) and validation cohort (n = 188) in a 7:3 ratio (using createDatapartition package). Nomograms for predicting overall survival (OS) and cancer-specific survival (CSS) were constructed by the development dataset and validated by the validation dataset. The study was approved by the institutional review board (IRB) of Qingdao municipal hospital, and the requirements for informed consent were waived off due to the retrospective design.

### Study Outcome

The primary endpoints for the nomograms were overall survival (OS) and cancer-specific survival (CSS). OS was defined as the time from initial diagnosis of PASC to death due to any cause or the date of the last follow-up; CSS was defined as the time from the first date of diagnosis until the occurrence of PASC-specific death.

### Statistical Analysis

The data were described as mean ± standard deviation (SD) for normally distributed continuous variables and median (interquartile range) for non-normally distributed data. Categorical variables were presented as frequencies and proportions. Quantitative data between development and validation cohorts were analyzed by Student’s *t*-test or the Mann–Whitney *U* test while qualitative data were compared by the χ^2^ test or Fisher’s exact probability test as appropriate. The survival curves were built by the Kaplan–Meier method and compared using the log-rank test. A penalized Cox’s proportional hazards model using the adaptive Least Absolute Shrinkage and Selection Operator (LASSO) was applied in the development cohort to identify predictive factors associated with OS and CSS (24–26). Based on the LASSO Cox regression model, nomograms of survival outcomes were formulated and internally validated by a bootstrap resampling process. The predictive accuracy of the nomograms was quantitatively measured by Harrell’s concordance index (C-index) and evaluated by calibration plots comparing nomogram-predicted estimates versus observed survival probability (27, 28). Time-dependent receiver operating characteristic (ROC) curves and area under curves were calculated to assess the models’ performance (29). The external verification of model performance was also assessed in the validation cohort by examining the discrimination and calibration. Additionally, a decision curve analysis (DCA) was carried out to evaluate the clinical utility of the prediction models by quantifying the net benefit of nomogram-assisted decisions. Individual risk scores were acquired according to the established nomograms (30). Risk stratification was based on an optimal threshold of risk score determined by surv_cutpoint function with maximally selected rank statistics in corresponding nomograms for OS or CSS. The cutoff values stratified patients into high-risk and low-risk groups and could provide the best discrepancy in survival analysis between risk groups. p value <0.05 was considered as statistically significant. All calculations were performed using R version 3.6.1.

## Results

### Baseline Characteristics

From 2000 to 2018, a total of 632 consecutive patients with PASC who met the inclusion criteria were retrospectively assessed and randomly divided into two groups by a ratio of 7:3 in our study. Patients’ baseline characteristics in the development (n = 444) and validation (n = 188) cohorts are presented in **Table 1**. Among the entire cohort, the majority of patients are 65 or older (63.4%), white (81.5%), and married (62.7%). Most tumors on presentation are with a diameter larger than 4 cm (58.5%) and a single tumor (95.4%). In addition, the most common tumor stage at presentation is regional defined by the SEER staging system. Of note, only 46.4% underwent surgery while more than half of the PASC patients (65.2%) received adjuvant chemotherapy. The clinical characteristics were well balanced between the two groups and the median OS and CSS in the development and validation cohorts were comparable, respectively.

**Table 1:** PASC patients characteristics in the study for the development and validation cohort.

Variables	Development cohort (n = 444)	Validation cohort (n = 188)	p value
Total	444	188	
Age			0.302
<65 years	168 (37.8%)	63 (33.5%)	
≥65 years	276 (62.2%)	125 (66.5%)	
Gender			0.344
Male	225 (50.7%)	103 (54.8%)	
Female	219 (49.3%)	85 (45.2%)	
Ethnicity			0.416
White	356 (80.2%)	159 (84.6%)	
Black	51 (11.5%)	16 (8.5%)	
Other	37 (8.3%)	13 (6.9%)	
Year of diagnosis			0.776
2000–2009	154 (34.7%)	63 (33.5%)	
2010–2018	290 (65.3%)	125 (66.5%)	
Marital status			0.971
Married	278 (62.6%)	118 (62.8%)	
Unmarried	166 (37.4%)	70 (37.2%)	
Tumor location			0.820
Head	211 (47.5%)	93 (49.5%)	
Body/tail	166 (37.4%)	70 (37.2%)	
Other	67 (15.1%)	25 (13.3%)	
Tumor size			0.193
≤2 cm	18 (4.0%)	3 (1.6%)	
2–4 cm	173 (39.0%)	68 (36.2%)	
>4 cm	253 (57.0%)	117 (62.2%)	
Tumor number			0.499
Single	422 (95.0%)	181 (96.3%)	
Multiple	22 (5.0%)	7 (3.7%)	
Lymph node status			0.966
Positive	178 (40.1%)	91 (48.4%)	
Negative	220 (49.5%)	77 (41.0%)	
Unknown	46 (10.4%)	20 (10.6%)	
Bone involvement			0.369
Yes	5 (1.1%)	5 (2.7%)	
No	280 (63.1%)	117 (62.2%)	
Unknown	159 (35.8%)	66 (35.1%)	
Liver involvement			0.940
Yes	92 (20.7%)	40 (21.3%)	
No	192 (43.2%)	83 (44.1%)	
Unknown	160 (36.1%)	65 (34.6%)	
Lung involvement			0.948
Yes	10 (2.3%)	5 (2.7%)	
No	273 (61.5%)	116 (61.7%)	
Unknown	161 (36.2%)	67 (35.6%)	
SEER stage			0.745
Localized	41 (9.2%)	17 (9.0%)	
Regional	212 (47.8%)	84 (44.7%)	
Distant	191 (43.0%)	87 (46.3%)	
Surgery			0.978
Yes	206 (46.4%)	87 (46.3%)	
No	238 (53.6%)	101 (53.7%)	
Radiation			0.677
Yes	56 (12.6%)	26 (13.8%)	
No	388 (87.4%)	162 (86.2%)	
Chemotherapy			0.919
Yes	290 (65.3%)	122 (64.9%)	
No	154 (34.7%)	66 (35.1%)	
Primary endpoint: OS, months			
Median (95% CI)	7.0 (6.19–7.82)	6.0 (4.46–7.54)	0.514†
Primary endpoint: CSS, months			
Median (95% CI)	7.0 (6.15–7.85)	7.0 (6.25–7.75)	0.515†

CI, confidence interval; ^†^Log-rank test.

### Feature Selection and Nomogram Construction

**Figure 1:**
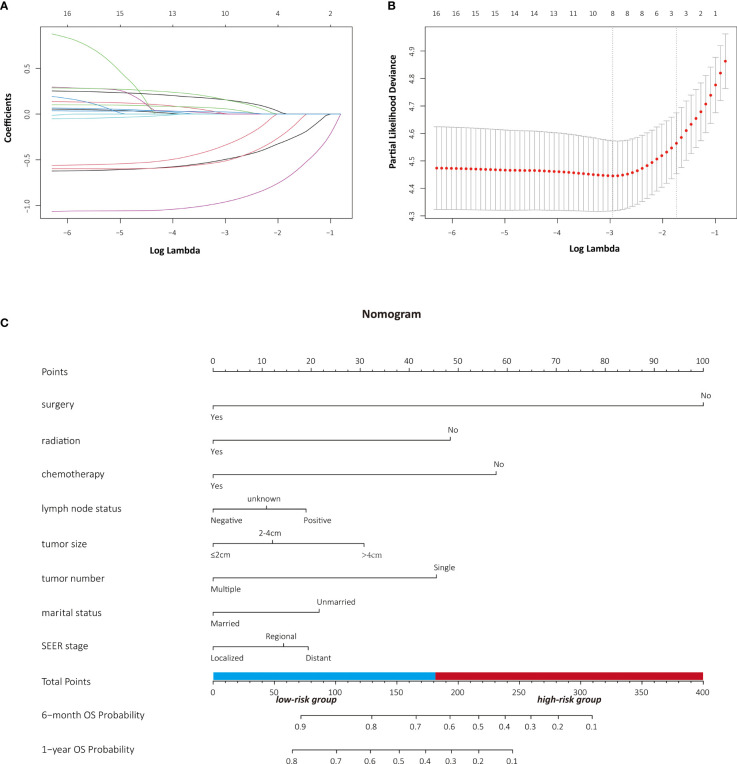
Identification of risk factors for OS in PASC patients with LASSO-Cox regression analysis **(A, B)**. A nomogram developed in the development cohort to predict overall survival of patients with PASC **(C)**.

**Figure 2:**
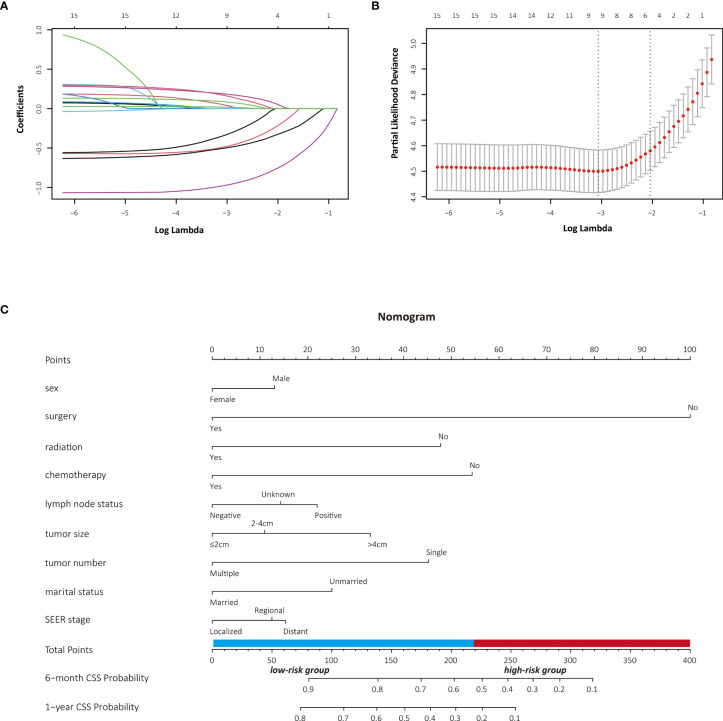
Identification of risk factors for CSS in PASC patients with LASSO-Cox regression analysis **(A, B)**. A nomogram developed in the development cohort to predict cancer-specific survival of patients with PASC **(C)**.

The LASSO Cox regression model was used to determine the optimal coefficient for each prognostic factor on the grounds of the minimum partial likelihood deviance. Coefficient profile plots were produced against the log (lambda) sequence (**Figure 1**). LASSO-penalized COX regression analysis-based minimum criteria using 10-fold cross-validation identified 8 independent predictors in the OS model: surgery, radiation, chemotherapy, lymph node status, tumor size, tumor number, marital status, and tumor stage (**Figure 1**). Risk factors selected in the CSS nomogram incorporated sex, surgery, radiation, chemotherapy, lymph node status, tumor size, tumor number, marital status, and tumor stage (**Figure 2**). All these selected candidate variables were then integrated in a multivariable Cox regression model to construct a nomogram-based model showing the probability of survival outcomes, respectively. (**Figures 1C**, **2C**)

### Assessment and Validation of the Nomogram for OS

**Figure 3:**
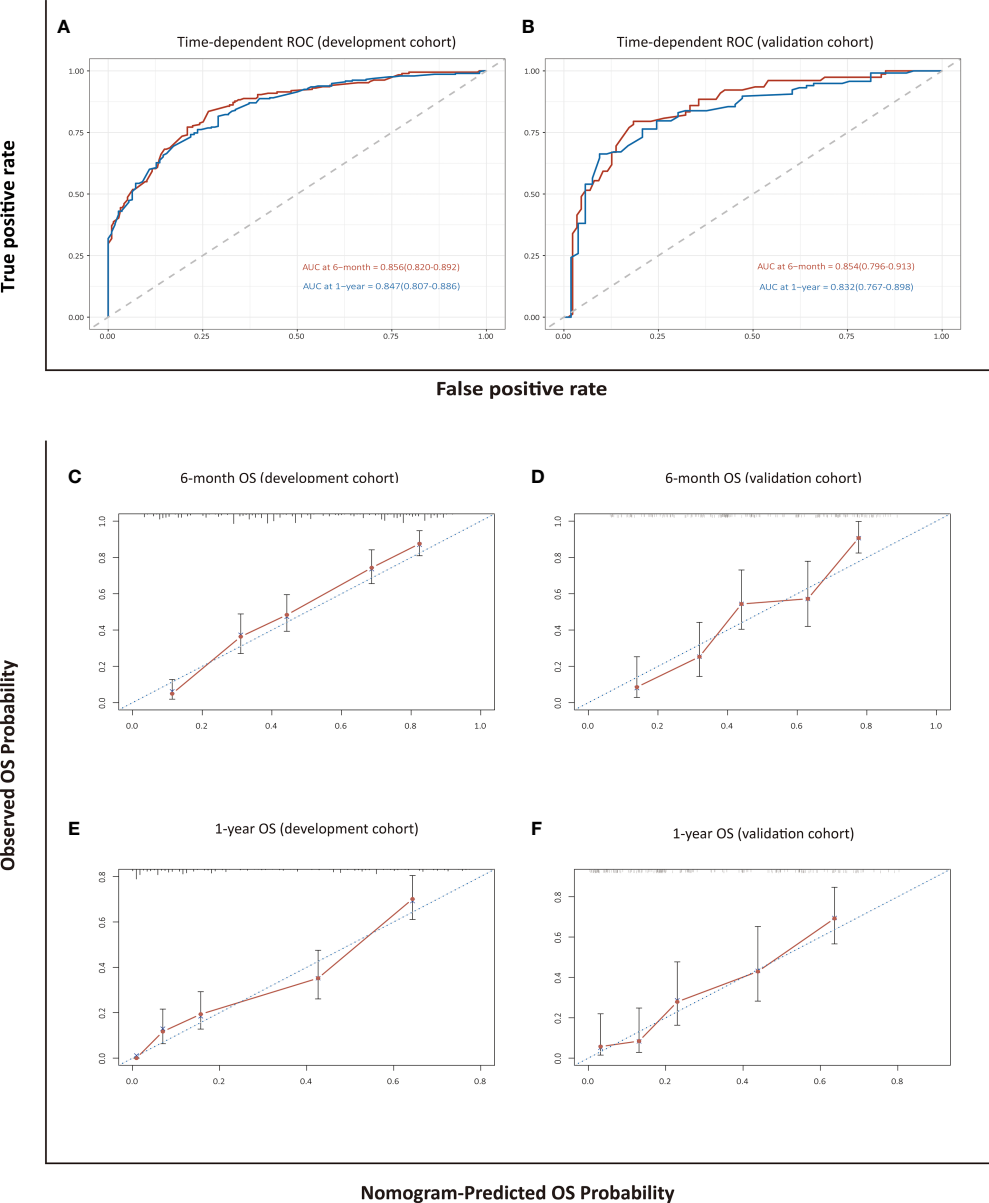
Assessment and validation of the nomogram-based model for OS. Time-dependent ROC curves **(A, B)** and calibration plots **(C–F)** of OS probabilities at 6 months and 1 year for internal and external validation.

The model for estimating 6-month and 1-year overall survival probability demonstrated a good prediction capability with a C-index of 0.762 (95% CI: 0.738–0.786) in the development cohort and 0.747 (95% CI: 0.710–0.785) in the validation cohort. The discrimination ability was also evaluated by time-dependent ROC curves and the area under the curves (AUCs). The LASSO-Cox regression model resulted in AUCs of 0.856 (95% CI: 0.820–0.892) and 0.847 (95% CI: 0.807–0.886) for 6-month and 1-year OS prediction, respectively, in the development group, and 0.854 (95% CI: 0.796–0.913) and 0.832 (95% CI: 0.767–0.898), respectively, in the validation group (**Figure 3**). The calibration curves in either the development or validation group showed good agreement between the nomogram-based prediction and observation in the probability of 6-month and 1-year survival (**Figure 3**).

### Assessment and Validation of the Nomogram for CSS

**Figure 4:**
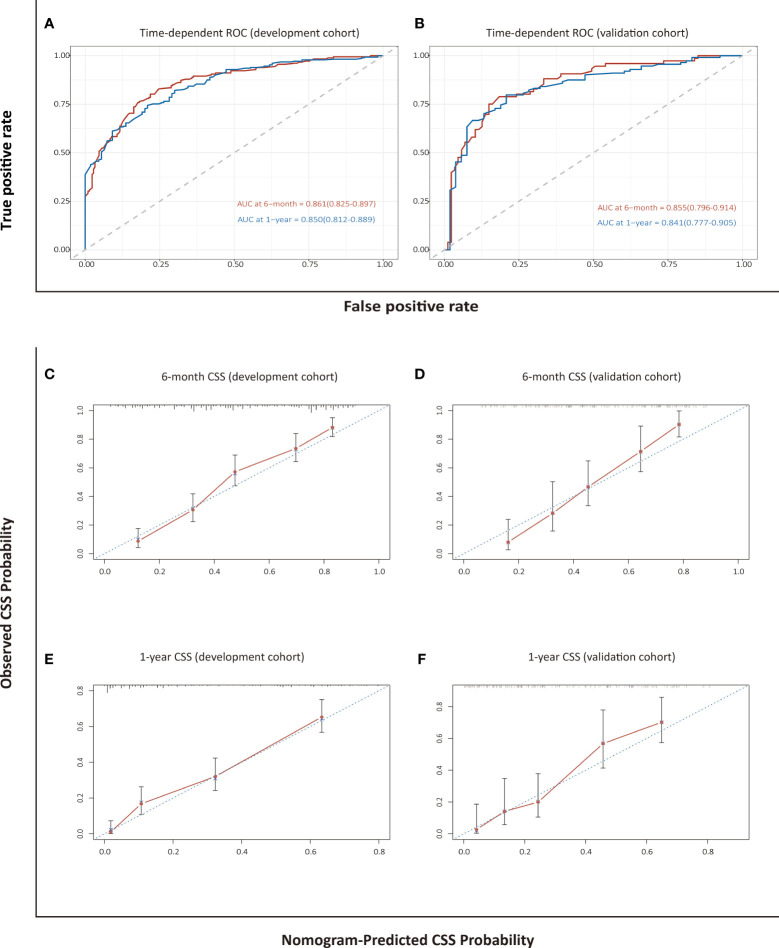
Assessment and validation of the nomogram-based model for CSS. Time-dependent ROC curves **(A, B)** and calibration plots **(C–F)** of CSS probabilities at 6 months and 1 year for internal and external validation.

The C-index of 0.789 (95% CI: 0.765–0.814) in the development cohort and 0.792 (95% CI: 0.755–0.830) in the validation cohort revealed a high discriminatory value for the nomogram-based model for CSS. The AUCs at 6 months and 1 year were 0.861 (0.825–0.897) and 0.850 (0.812–0.889), respectively, in the development cohort, and 0.855 (0.796–0.914) and 0.841 (0.777–0.905), respectively, in the validation cohort, which indicated high predictive accuracy (**Figure 4**). Calibration plots showed that the established nomogram for CSS performed well in predicting the probability of 6-month and 1-year survival (**Figure 4**).

### Decision Curve Analysis

**Figure 5:**
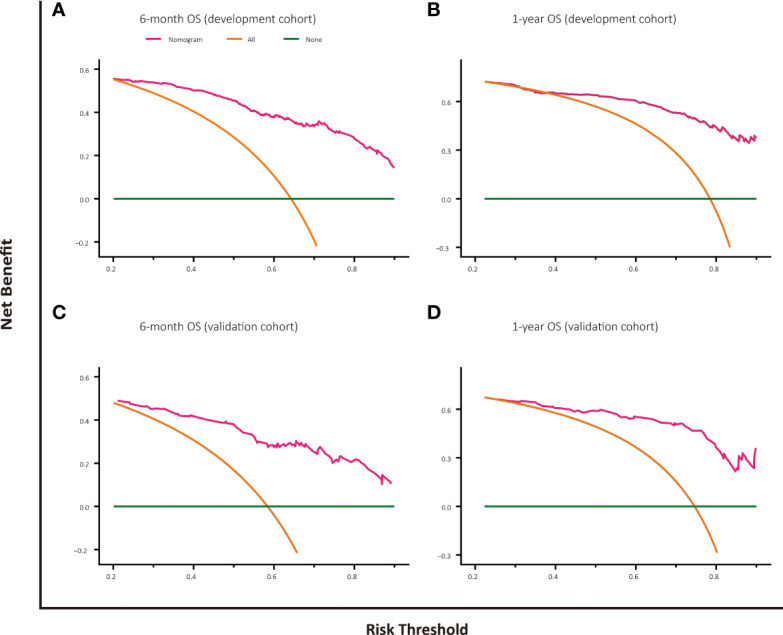
Decision curve analysis for OS prediction at 6 months and 1 year in the development and validation cohorts. **(A)** DCA for 6-month OS predction in the development cohort. **(B)** DCA for 1-year OS prediction in the development cohort. **(C)** DCA for 6-month OS prediction in the validation cohort. **(D)** DCA for 1-year OS prediction in the validation cohort.

**Figure 6:**
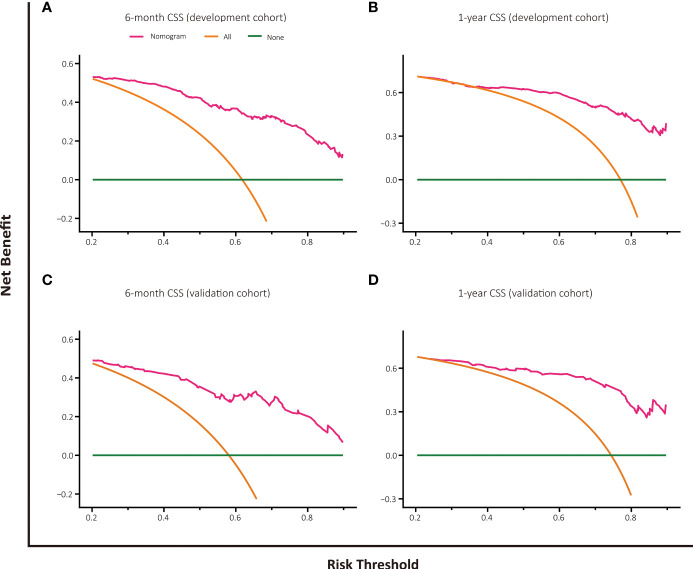
Decision curve analysis for CSS prediction at 6 months and 1 year in the development and validation cohorts. **(A)** DCA for 6-month CSS predction in the development cohort. **(B)** DCA for 1-year CSS prediction in the development cohort. **(C)** DCA for 6-month CSS prediction in the validation cohort. **(D)** DCA for 1-year CSS prediction in the validation cohort.

The decision curve analysis of nomogram-based models for OS or CSS both displayed good clinical utility and favorable predictive efficiency in prediction of 6-month as well as 1-year survival with a wide range of beneficial threshold probabilities (**Figures 5**, **6**).

### Risk Stratification Based on the Nomogram

**Figure 7:**
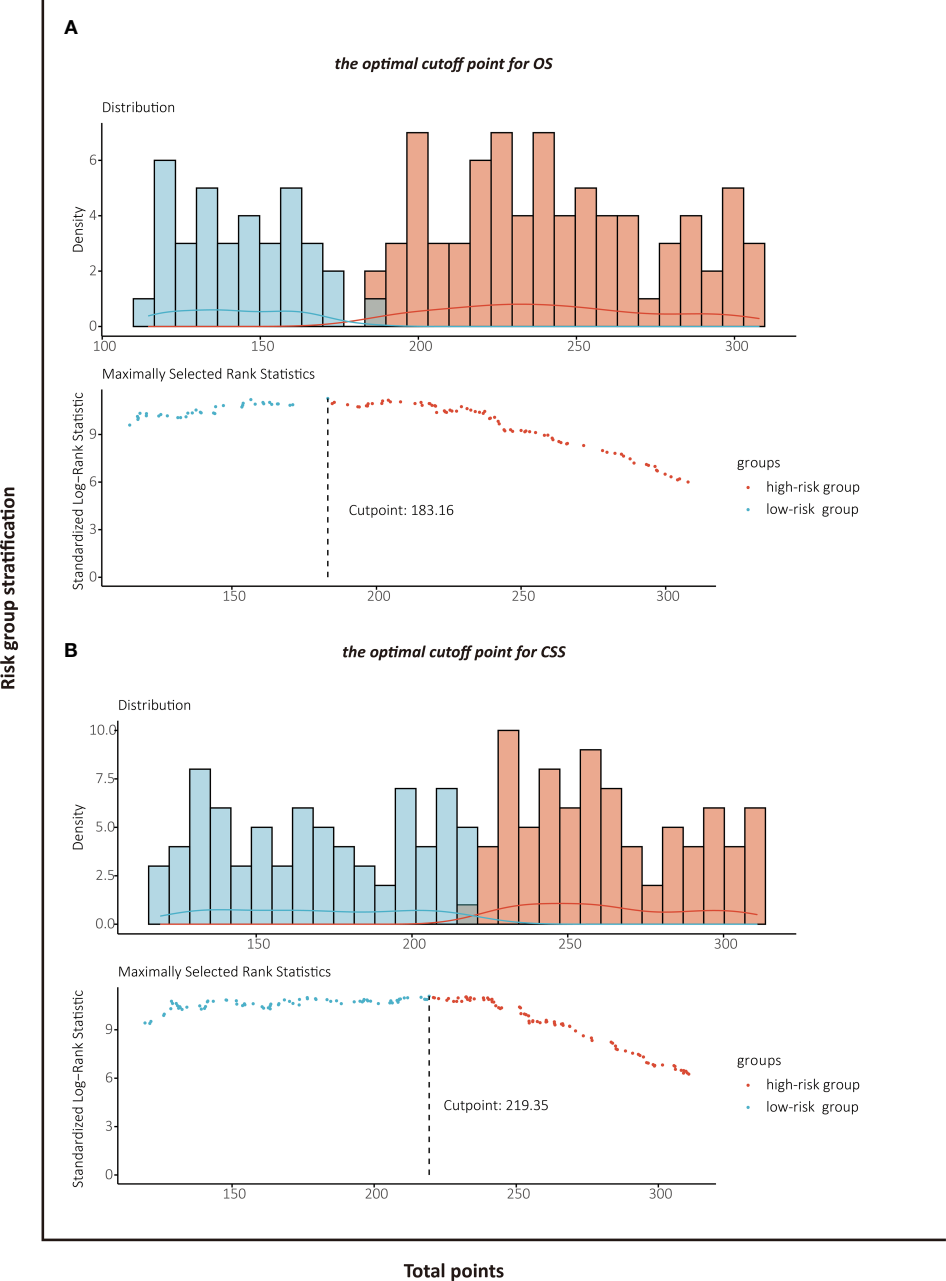
Identification of the optimal cutoff points for risk scores in OS prediction model **(A)** and CSS prediction model **(B)** by survminer package.

**Figure 8:**
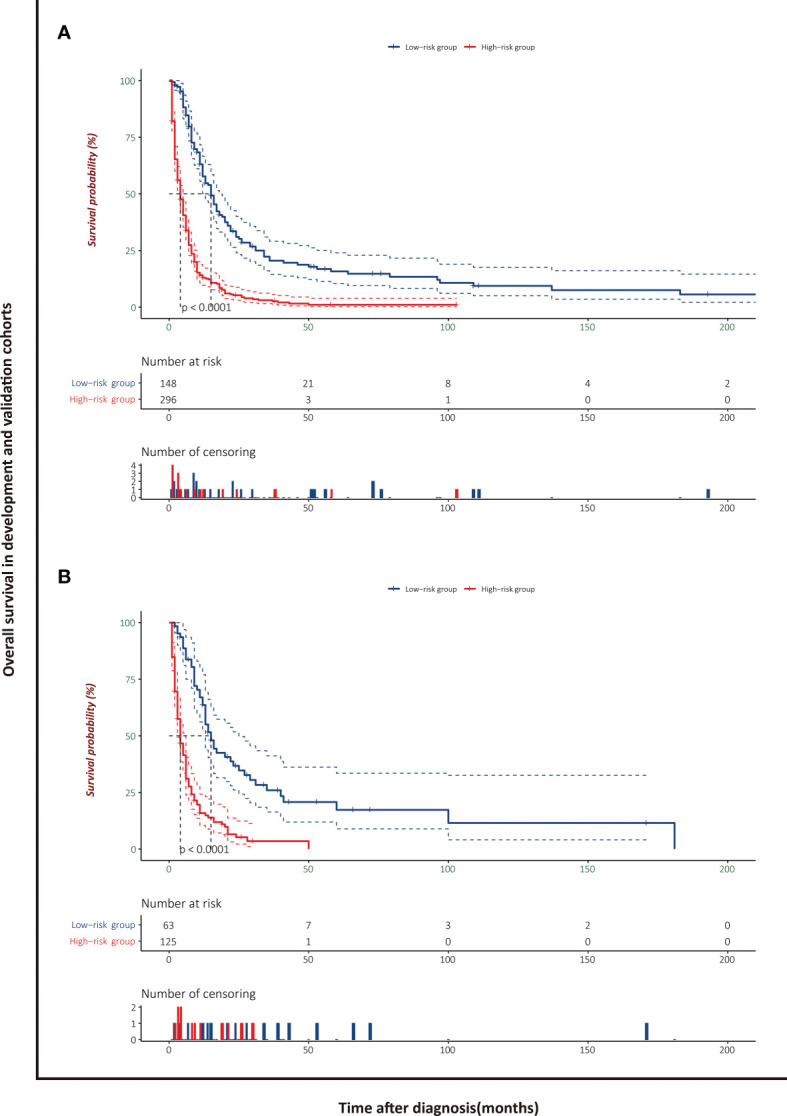
Overall survival analysis of patients with PASC stratified by risk scores in the development cohort **(A)** and validation cohort **(B)**.

**Figure 9:**
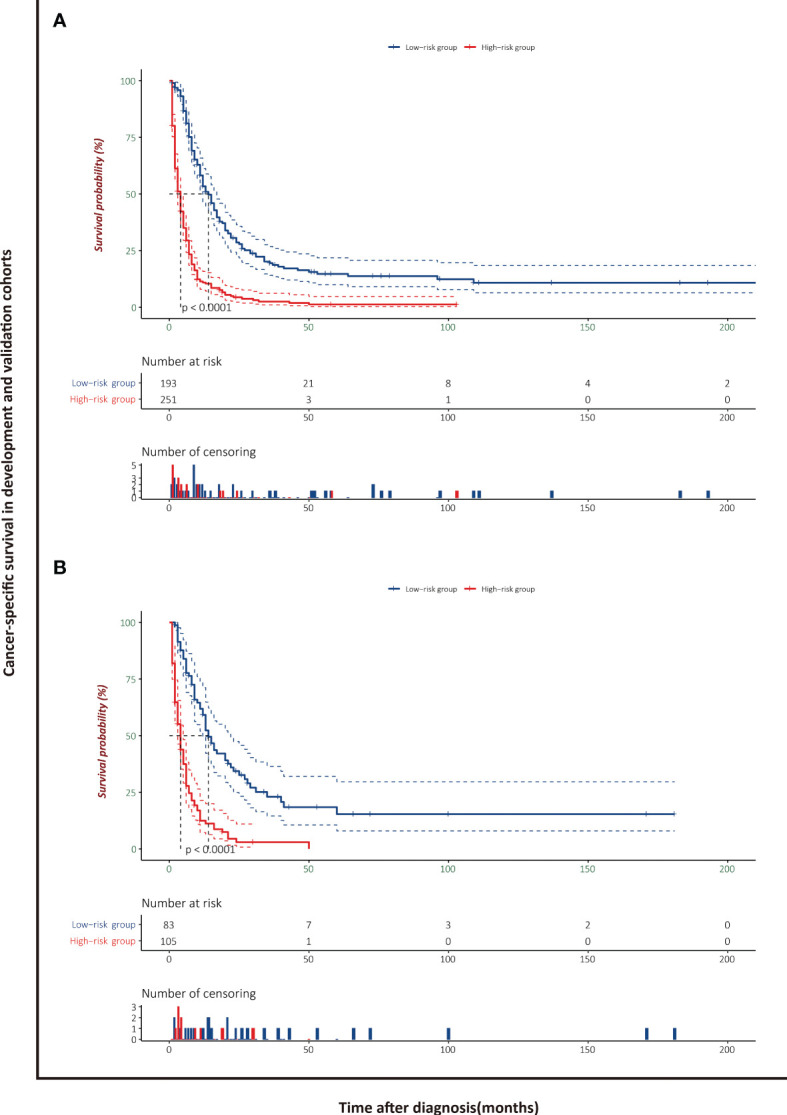
Cancer-specific survival analysis of patients with PASC stratified by risk scores in the development cohort **(A)** and validation cohort **(B)**.

The patients stratified by the LASSO-Cox regression models were classified into high-risk and low-risk groups according to the optimal cutoff points for risk scores calculated by survminer package (183.16 points for the OS model and 219.35 points for the CSS model) (**Figure 7**). Clinicopathological characteristics of the risk groups for patients are listed in **Table 2**. Survival curves by risk groups were built by the Kaplan–Meier method and compared using the log-rank test. All the survival curves exhibited great discrimination between the two groups (p < 0.001) (**Figures 8**, **9**).

**Table 2:** PASC patients characteristics stratified by risk scores.

Variables	Risk groups for OS			Risk groups for CSS		
	High-risk group (n = 421)	Low-risk group (n = 211)	p value	High-risk group (n = 356)	Low-risk group (n = 276)	p value
Age			0.057			0.129
<65 years	143 (34.0%)	88 (41.7%)		121 (34.0%)	110 (39.9%)	
≥65 years	278 (66.0%)	123 (58.3%)		235 (66.0%)	166 (60.1%)	
Gender			0.674			0.316
Male	216 (51.3%)	112 (53.1%)		165 (46.3%)	137 (49.6%)	
Female	205 (48.7%)	99 (46.9%)		191 (53.7%)	139 (50.4%)	
Ethnicity			0.505			0.813
White	344 (81.7%)	171 (81.0%)		292 (82.0%)	223 (80.8%)	
Black	47 (11.2%)	20 (9.5%)		38 (10.7%)	29 (10.5%)	
Other	30 (7.1%)	20 (9.5%)		26 (7.3%)	24 (8.7%)	
Year of diagnosis			0.664			0.421
2000–2009	147 (34.9%)	70 (33.2%)		127 (35.7%)	90 (32.6%)	
2010–2018	274 (65.1%)	141 (66.8%)		229 (64.3%)	186 (67.4%)	
Marital status			**0.001**			**0.001**
Married	237 (56.3%)	159 (75.4%)		196 (55.1%)	200 (72.5%)	
Unmarried	184 (43.7%)	52 (24.6%)		160 (44.9%)	76 (27.5%)	
Tumor location			0.055			**0.036**
Head	191 (45.4%)	113 (53.6%)		159 (44.7%)	145 (52.5%)	
Body/tail	160 (38.0%)	76 (36.0%)		135 (37.9%)	101 (36.6%)	
Other	70 (16.6%)	22 (10.4%)		62 (17.4%)	30 (10.9%)	
Tumor size			**0.001**			**0.001**
≤2 cm	13 (3.1%)	8 (3.8%)		9 (2.5%)	12 (4.3%)	
2–4 cm	131 (31.1%)	110 (52.1%)		100 (28.1%)	141 (51.1%)	
>4 cm	277 (65.8%)	93 (44.1%)		247 (69.4%)	123 (44.6%)	
Tumor number			0.082			**0.015**
Single	406 (96.4%)	197 (93.4%)		346 (97.2%)	257 (93.1%)	
Multiple	15 (3.6%)	14 (6.6%)		10 (2.8%)	19 (6.9%)	
Lymph node status			**0.001**			**0.001**
Positive	136 (32.3%)	119 (56.4%)		108 (30.3%)	147 (53.3%)	
Negative	222 (52.7%)	89 (42.2%)		185 (52.0%)	126 (45.6%)	
Unknown	63 (15.0%)	3 (1.4%)		63 (17.7%)	3 (1.1%)	
Bone involvement			0.050			**0.006**
Yes	10 (2.4%)	0		10 (2.8%)	0	
No	257 (61.0%)	140 (66.4%)		212 (59.6%)	185 (67.0%)	
Unknown	154 (36.6%)	71 (33.6%)		134 (37.6%)	91 (33.0%)	
Liver involvement			**0.001**			**0.001**
Yes	125 (29.7%)	7 (3.3%)		119 (33.4%)	13 (4.7%)	
No	142 (33.7%)	133 (63.1%)		103 (28.9%)	172 (62.3%)	
Unknown	154 (36.6%)	71 (33.6%)		134 (37.7%)	91 (33.0%)	
Lung involvement			**0.010**			**0.003**
Yes	15 (3.6%)	0		14 (3.9%)	1 (0.3%)	
No	249 (59.1%)	140 (66.4%)		205 (57.6%)	184 (66.7%)	
Unknown	157 (37.3%)	71 (33.6%)		137 (38.5%)	91 (33.0%)	
SEER stage			**0.001**			**0.001**
Localized	33 (7.8%)	25 (11.8%)		25 (7.0%)	33 (12.0%)	
Regional	147 (34.9%)	149 (70.7%)		106 (29.8%)	190 (68.8%)	
Distant	241 (57.3%)	37 (17.5%)		225 (63.2%)	53 (19.2%)	
Surgery			**0.001**			**0.001**
Yes	82 (19.5%)	211 (100%)		34 (9.6%)	259 (93.8%)	
No	339 (80.5%)	0		322 (90.4%)	17 (6.2%)	
Radiation			**0.001**			**0.001**
Yes	6 (1.4%)	76 (36.0%)		1 (0.3%)	81 (29.3%)	
No	415 (98.6%)	135 (64.0%)		355 (99.7%)	195 (70.7%)	
Chemotherapy			**0.001**			**0.001**
Yes	216 (51.3%)	196 (92.9%)		190 (53.4%)	222 (80.4%)	
No	205 (48.7%)	15 (7.1%)		166 (46.6%)	54 (19.6%)	
Primary endpoint: OS, months						
Median (95% CI)	4.0 (3.38–4.62)	15.0 (12.96–17.04)	**0.001**†	4.0 (3.48–4.52)	13.0 (11.19-14.81)	**0.001**†
Primary endpoint: CSS, months						
Median (95% CI)	4.0 (3.37–4.63)	15.0 (12.77–17.23)	**0.001**†	4.0 (3.47–4.53)	14.0 (11.96-16.04)	**0.001**†

†Log-rank test; Bold values indicate p < 0.05.

## Discussion

In this retrospective analysis and larger population-based study, novel nomograms for prediction of OS and CSS were developed and validated. According to the established models, patients with pancreatic adenosquamous carcinoma (PASC) were separated into high-risk and low-risk groups, which could significantly improve the prediction capabilities of long-term outcomes and provide appropriate decision-making guidance. Of note, the selection of significant predictors that entered into the construction of nomograms was on the basis of a least absolute shrinkage and selection operator (LASSO)-Cox regression model. Meanwhile, the discrimination, calibration, and utility assessment in the current study demonstrated that the nomogram-based models performed well in prognostic prediction.

Of the exocrine pancreatic cancers, the most common type is pancreatic ductal carcinoma (PDAC); in contrast, the pancreatic adenosquamous carcinoma (PASC) characterized by a histological admixture of glandular epithelium and malignant squamous epithelium remains an extremely rare subtype. However, the diagnostic criteria of PASC regarding to the percentage of squamous component is in dispute. In general, the squamous differentiation should account for more than 30% of the neoplasm to qualify as PASC. Nevertheless, some investigators have questioned the strict criteria and argued that the evaluation of the tumor proportion is too subjective. Instead, pancreatic cancers with the presence of any degree of malignant squamous composition should be defined as PASC (12). To date, the etiology of histogenesis of PASC was still unclear. The hypotheses to explain this phenomenon include the disputable collision tumor hypothesis, malignant squamous metaplasia of the ductal adenocarcinoma, and development from a progenitor cell (31–36). As a result, the epidemiology and clinical course of PASC remain poorly understood.

As shown in the mapped nomograms, chemotherapy that spread through the full range of point axis was considered as the most prominent prognostic factor either for OS or for CSS, followed by surgical intervention. Among the low-risk groups for OS or CSS, more than 93% of PASC patients underwent surgical resection, while less than 20% in the high-risk groups. With respect to adjuvant chemotherapy, nearly all of the patients received this treatment in low-risk groups. The estimated OS and CSS differ significantly between the two different risk groups, respectively. Surgery serves as the mainstay of curative treatment in resectable and borderline resectable disease, while adjuvant chemotherapy remains the primary treatment modality in locally advanced or distant metastases patients and can increase the R0 resection rate (37–39). Patients with pancreatic cancer were mainly diagnosed with advanced stage at the time of diagnosis owing to asymptomatic or vague symptoms in the early phase, ultimately resulting in a decreased chance of resection and thus a poor prognosis. The similar clinical presentations to PDAC such as abdominal pain, weight loss, and jaundice make it difficult to distinguish PASC from conventional PDAC. Previous trials have confirmed the survival benefit of surgery and adjuvant chemotherapy in patients with PASC (12, 40, 41). In a large-scale study with matched-pair analysis to evaluate the OS between PASC and PDAC, although the former was frequently inclined to characterize with more aggressive behaviors, the 5-year survival probabilities were comparable after surgical resection (15). Another study based on the National Cancer Database by Hester et al. reported that surgery in resected stage patients with PASC was associated with overall survival benefits (18). In a retrospective study of the SEER database including 415 patients who were diagnosed with PASC, Boyd et al. demonstrated that the surgical patients were combined with a survival advantage compared to non-surgical patients (11). Standard chemotherapy regimens for PDAC such as FOLFIRINOX, gemcitabine, and capecitabine have been proven of effectiveness, and some novel strategies are underway (42–44). However, there are limited data regarding the regimens related to PASC. In our study, the chemotherapy did show significant predictive strength for OS and CSS in PASC patients, which is in keeping with the results of some other research (45, 46). During daily clinical practice, the PASC patients were more likely to be treated on the grounds of treatment strategies for PDAC. In other words, there is still much pertaining to adjuvant chemotherapy in PASC that need to be dealt with.

These two nomograms incorporated key indicators selected by the LASSO-Cox regression procedure based on a large-population database were developed to predict prognosis in patients with PASC. Unlike traditional Cox regression methods, the penalized variable selection method improved the predictive performance and interpretability of model by the shrinkage property. It is noteworthy that these two nomograms, one for OS and the other for CSS, were developed to apply to all patients with initial diagnosis of PASC. One strength of our study is that the nomogram-based models conducted successful internal and external validation illustrated by good discrimination and calibration.

To the best of our knowledge, there are relatively few models available for predicting survival outcomes of patients with PASC. As a leading cause of cancer-related death worldwide, the exact prediction of survival results in patients with pancreatic cancer is of utmost interest to both physicians and patients. However, the vast majority of previous research focused on patients with pancreatic ductal carcinoma (PDAC), which accounts for more than 90% of exocrine pancreatic cancer (8, 47). In contrast to most of the published literature, the main focus of our study is the survival outcomes in patients with PASC. In a retrospective study that evaluates the association between radiological features and survival in PASC patients, the authors found that the characteristic features in PASC may be useful in predicting the prognosis (48). However, this study only included 26 patients. Another strength of our study, therefore, is that the nomograms were built based on a large series of PASC patients (n = 444) which made the results become more reproducible and stable. In view of that all predictive parameters in the models are existing clinical data, it is convenient to predict individualized survival in patients with PASC.

The present study is of vital clinical significance in that nomograms built in the development cohort can be used to estimate and refine individualized prognosis of patients with PASC. Moreover, patients might be stratified into high-risk and low-risk groups according to the risk scores calculated by the nomograms, which could aid clinical decision-making and provide guidance for clinicians.

Our study had several limitations. Firstly, the inherent biases with a retrospective study could not be thoroughly eliminated. Secondly, data regarding the pathological parameters, tumor markers, treatment details, and other survival outcomes in the database precluded the further analysis. Thirdly, an external validation in a validation set from other sources would be more appropriate to determine the prediction models’ reproducibility and generalizability to different patients. Fourthly, considering the risk factors selected in our models, the application of our nomograms might be restricted for predicting prognosis before surgery. Finally, the nomograms constructed in our study still need to be validated the reliability and utility by prospective trial data.

## Conclusion

In conclusion, nomogram-based models to evaluate personalized overall survival and cancer-specific survival in patients with pancreatic adenosquamous carcinoma were developed and well validated with outstanding predictive accuracy. These easy-to-use tools will be useful methods to calculate individualized estimate of survival, assist in risk stratification, and aid clinical decision-making.

## Data Availability Statement

The datasets presented in this study can be found in online repositories. The names of the repository/repositories and accession number(s) can be found in the following: https://seer.cancer.gov/data.

## Author Contributions

GS and PZ contributed to the conception and analyzed the data. ZY designed the study and wrote the manuscript. All authors contributed to the article and approved the submitted version.

## Conflict of Interest

The authors declare that the research was conducted in the absence of any commercial or financial relationships that could be construed as a potential conflict of interest.

## Publisher’s Note

All claims expressed in this article are solely those of the authors and do not necessarily represent those of their affiliated organizations, or those of the publisher, the editors and the reviewers. Any product that may be evaluated in this article, or claim that may be made by its manufacturer, is not guaranteed or endorsed by the publisher.
